# New Bioactive Peptides Identified from a Tilapia Byproduct Hydrolysate Exerting Effects on DPP-IV Activity and Intestinal Hormones Regulation after Canine Gastrointestinal Simulated Digestion

**DOI:** 10.3390/molecules26010136

**Published:** 2020-12-30

**Authors:** Sandy Theysgeur, Benoit Cudennec, Barbara Deracinois, Claire Perrin, Isabelle Guiller, Anne Lepoudère, Christophe Flahaut, Rozenn Ravallec

**Affiliations:** 1 UMR-T 1158, BioEcoAgro, University of Lille, F-59000 Lille, France; sandy.th@hotmail.fr (S.T.); barbara.deracinois@univ-lille.fr (B.D.); christophe.flahaut@univ-artois.fr (C.F.); 2 Diana Pet Food, F-56250 Elven, France; cperrin@diana-petfood.com (C.P.); iguiller@diana-petfood.com (I.G.); alepoudere@diana-petfood.com (A.L.); 3 UMR Transfrontalière BioEcoAgro N° 1158, University of Artois, F-62000 Arras, France

**Keywords:** bioactive peptides, in vitro gastrointestinal digestion, fish byproduct hydrolysate, cholecystokinin, glucagon-like peptide 1, DPP-IV inhibitory peptides

## Abstract

Like their owners, dogs and cats are more and more affected by overweight and obesity-related problems and interest in functional pet foods is growing sharply. Through numerous studies, fish protein hydrolysates have proved their worth to prevent and manage obesity-related comorbidities like diabetes. In this work, a human in vitro static simulated gastrointestinal digestion model was adapted to the dog which allowed us to demonstrate the promising effects of a tilapia byproduct hydrolysate on the regulation of food intake and glucose metabolism. Promising effects on intestinal hormones secretion and dipeptidyl peptidase IV (DPP-IV) inhibitory activity were evidenced. We identify new bioactive peptides able to stimulate cholecystokinin (CCK) and glucagon-like peptide 1 (GLP-1) secretions, and to inhibit the DPP-IV activity after a transport study through a Caco-2 cell monolayer.

## 1. Introduction

The world population is projected to rise by 2 billion in the next 30 years, reaching 9.7 billion in 2050 [1]. This growth implies in an increase in food consumption, and the protein demand will significantly grow as a result of socio-economic changes, increased urbanization, rising incomes and the recognition of the role of protein in healthy diets. Dietary protein production exerts a high environmental impact, particularly for animal-derived protein, which causes high greenhouse gas emissions, land-use changes linked to an important terrestrial biodiversity loss and a high-water demand [2]. In this context, there is an important need to valorize and to better characterize dietary protein derived-byproducts to optimize their use and to answer the worldwide growing protein demand. For instance, in 2018, about 25% of the 178 million tons of global fish and shellfish production were lost or wasted [3]. The valorization processes of fish high-quality protein byproducts will partially address these issues by offering a renewable alternative, whilst creating added value in numerous domains such as in functional food or the pet food industry. The global pet food market was valued at USD 103.5 billion in 2016 in which the segment of healthcare and nutritional supplements shared 5%. In parallel, overweight and obesity and their associated chronic diseases such as type 2 diabetes mellitus (T2DM) are growing at a worrying rate around the globe. In 2016, 650 million adults were obese, amongst 1.9 billion overweight persons. In 2019, the prevalence of T2DM was estimated at 417 million and the projection for 2045 is about 630 million [4].

Like humans, companion dogs and cats are affected by overweight and obesity comorbidities such as diabetes and cancers, leading to impaired health and reduced life span. Depending on breeds and the methodology used to evaluate health status, overweight and obesity prevalence was estimated between 19.7% and 59.3% in dogs and between 7% to 63% in cats. This situation is mainly due to excessive food offer and related calorie intake, as pet owners do not follow nutritional guidance, and to a loss of physical exercise leading to the overweight-derived problems mentioned above but also to skin disorders, respiratory and locomotor diseases [5,6].

Dietary protein digestion produces the release of peptides and free amino acids, which regulate short term food intake. Protein-digested products stimulate the secretion of satiety signals via the “intestinal sensing” phenomenon, a nutrient recognition on the apical side of the enteroendocrine cells (EECs) [7,8]. Nevertheless, mechanisms that lead to gut hormones secretion by EECs after peptide and amino acid intestinal sensing remain unclear [9]. The two well-known intestinal anorexigenic hormones, cholecystokinin (CCK) and glucagon-like peptide-1 (GLP-1), exert their satiating effect via different pathways. GLP-1 also plays a significant role in glucose metabolism by regulating blood glucose via its incretin action [10]. After its secretion by EECs following a meal, circulating level of GLP-1 increases but has a short half-life because it is inactivated by the dipeptidyl peptidase 4 enzyme (DPP-IV) which is a serine protease present in a soluble form (plasma, urine, amniotic fluid) as well as in a membranous form in a wide type of cells and organs like the intestine, kidney or liver [11]. Hence, GLP-1 agonists and DPP-IV inhibitors have been targeted to treat insulin resistance occurring in T2DM [12]. These past few years, numerous dietary protein-derived peptides have been identified as DPP-IV inhibitors. Albeit they are less potent than drugs, they have shown a rising interest as a natural alternative to chemical DPP-IV inhibitors which harbor important side effects [13].

Fish protein- and hydrolysate-derived bioactive peptides have been identified to exert many in vitro and in vivo bioactivities, suggesting promising health benefits via several pathways involved in hypertension, obesity, inflammation, or in the regulation of glucose homeostasis in metabolic disorders [14]. Indeed, numerous studies have shown, in vitro as well as in vivo, the beneficial effects of fish hydrolysates on food intake regulation by the stimulation of the gut hormones secretion, in particular CCK and GLP-1 [15,16,17,18]. Moreover, fish hydrolysates could improve glucose homeostasis by increasing plasma GLP-1, gastric inhibitory peptide, also known as glucose-dependent insulinotropic polypeptide (GIP) increasing insulin secretion and lowering blood glucose [19,20] but also by reducing DPP-IV activity in vitro and in vivo [21,22,23].

The Nile tilapia (*Oreochromis niloticus*) is the third most produced species in the world for which the production was more than 4500 thousand tons representing 8.3% of the world aquaculture production in 2018. These days the development of fish processing has resulted in growing quantities of byproducts which can represent more than 70 percent of the processed fish [3].

In this work, we investigated whether a tilapia fish byproduct protein hydrolysate (FBPH) compared to its raw material (FBP), both submitted to an in vitro simulated gastrointestinal digestion (SGID), could stimulate gut hormones secretion in EECs and inhibit intestinal DPP-IV activity. This work aimed to future pet applications, so the consensual human SGID INFOGEST protocol was adapted to the dog digestion [24].

## 2. Results

### 2.1. Peptide Profile Modifications during SGID

**Figure 1:**
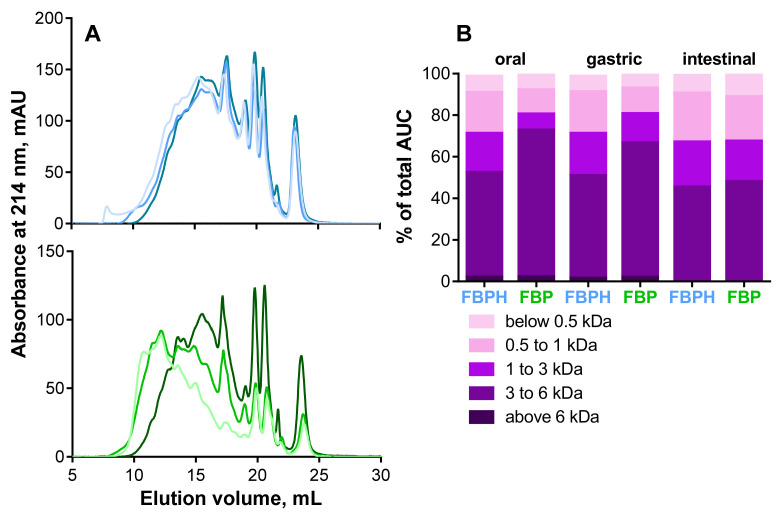
Peptide profiles and peptide molecular weight (MW) distributions. (**A**) Peptide profiles were obtained by size exclusion chromatography-fast protein liquid chromatography (SEC-FPLC): FBPH (blue curves), FBP (green curves); oral (light colored curves), gastric (colored curves) and intestinal (dark colored curves) digests. (**B**) The MW distribution of peptides in the different SGID compartments, expressed in percentage of total area under the curve (AUC), was calculated from the linear regression relationship which correlates the Log of known MW standard peptides and the elution volume.

To characterize and compare the impact of SGID on elution profiles and peptide apparent molecular weight (MW) distributions, FBPH and FBP, gastric and intestinal digests were submitted to SEC-FPLC. The oral digest both for FBPH and FBP serves as a referent peptide elution profile. The gastric and intestinal SGID phases did not extensively modify the shape of the peptide elution profiles of FBPH. In contrast, for FBP, significant modifications occurred during the SGID, as illustrated by the curve shift towards lower MW between the oral and the intestinal phases (Figure 1A). Besides, the MW distribution showed that the impact of the SGID was less significant for FBPH than for FBP. Thus, the proportions of high MW peptides (above 3 kDa) in oral, gastric and intestinal digests represented 72.8%, 66.6% and 48.1% for FBP and 52.4%, 50.9%, 45.4% for FBPH, respectively (Figure 1B). Moreover, in the intestinal phase, high MW peptides (above 6 kDa) disappeared entirely for both FBP and FBPH digests. The same phenomenon was observed for small MW peptides (below 1 kDa) for which the proportion increased during the SGID in a less extent manner for FBPH than for FBP. Indeed, their proportions in oral, gastric and intestinal digests were 27.5%, 27.5%, and 32.0% for FBPH and 19.0%, 19.0% and 32.9% for FBP, respectively. Despite slight differences, at the end of the SGID, the MW distribution profiles of FBP and FBPH were similar.

### 2.2. CCK and GLP-1 Secretion Induced by FBPH and FBP Digests

**Figure 2:**
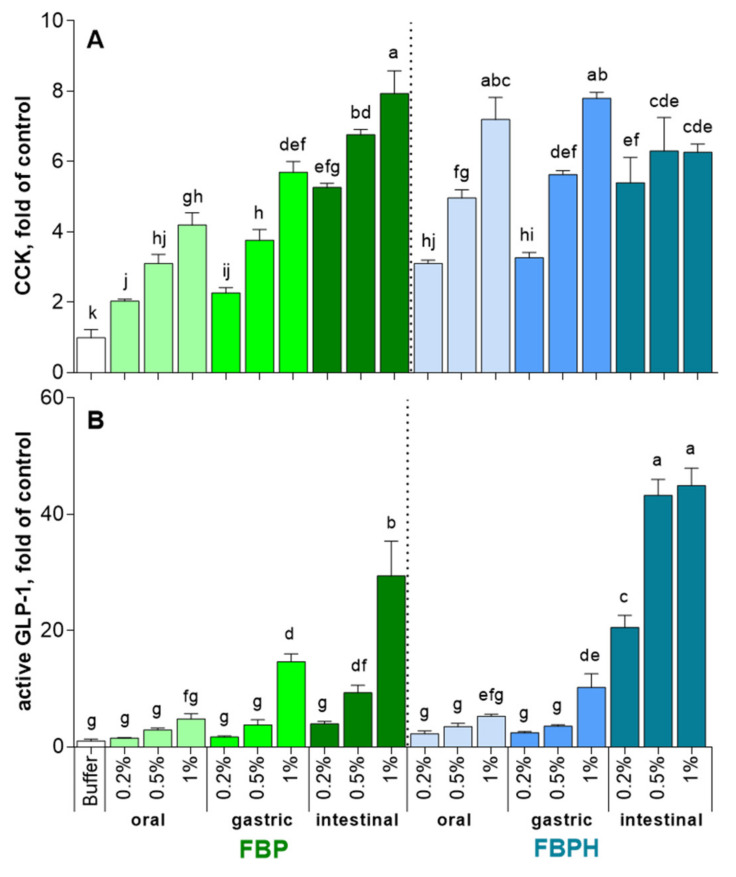
FBPH and FBP induction of intestinal hormones release during simulated GI digestion. The amounts of intestinal hormones released in STC-1 cells in the supernatants after 2 h of contact with the FBP and FBPH digests (2; 5 and 10 mg mL^−1^ *w*/*v*) were determined by radioimmunoassay for CCK (**A**) and active GLP-1 (**B**). Values are means ± SD and are expressed in fold of control (buffer). Means without a common letter within the same graph are significantly different (*p* < 0.05) using one-way ANOVA following by Tukey post-hoc test for pairwise comparisons.

The STC-1 cells exposure to increasing concentrations (2, 5 and 10 mg mL^−1^ *w*/*v*) of oral and gastric digests of FBP and FBPH induced a dose-dependent increase in CCK release (Figure 2A). At the higher concentration tested (10 mg mL^−1^ *w*/*v*), the FBPH contact led to a better stimulating effect with 7.2 ± 0.6 and 7.8 ± 0.2-fold of control (FOC) while the amounts of CCK obtained after FBP contact were 4.2 ± 0.4 and 5.7 ± 0.3 FOC for oral and gastric samples, respectively. Conversely, the intestinal digest of FBP highly stimulated the secretion of CCK (7.9 ± 0.7 FOC). The digestive process enhanced the ability of FBP to stimulate CCK secretion. Conversely, for FBPH, sole the intestinal phase of the SGID led to a slight diminution in CCK secretion in STC-1 cells at 10 mg mL (*w*/*v*).

The effects of oral and gastric digests of FBP and FBPH on GLP-1 secretion stimulation were equivalent (Figure 2B). Thus, only the gastric 10 mg mL (*w*/*v*) dose induced a significant increase of GLP-1 secretion with 14.6 ± 1.4 and 10.2 ± 2.3 FOC for FBP and FBPH, respectively. After the intestinal phase, the stimulating effect of FBPH digest on GLP-1 secretion was highly enhanced and led to 20.6 ± 2.1, 43.3 ± 2.7 and 45 ± 3.0 FOC for 2, 5 and 10 mg mL (*w*/*v*) concentrations, respectively. The effect of the SGID intestinal phase on the ability of FBP to enhance the stimulation of GLP-1 secretion was weaker. Indeed, the results were significant only for 5 and 10 mg mL (*w*/*v*) doses with recovered GLP-1 secretions of 9.3 ± 1.3 and 29.4 ± 6.0 FOC, respectively.

### 2.3. Intestinal DPP-IV Inhibition Activity of FBPH and FBP Digests

**Figure 3:**
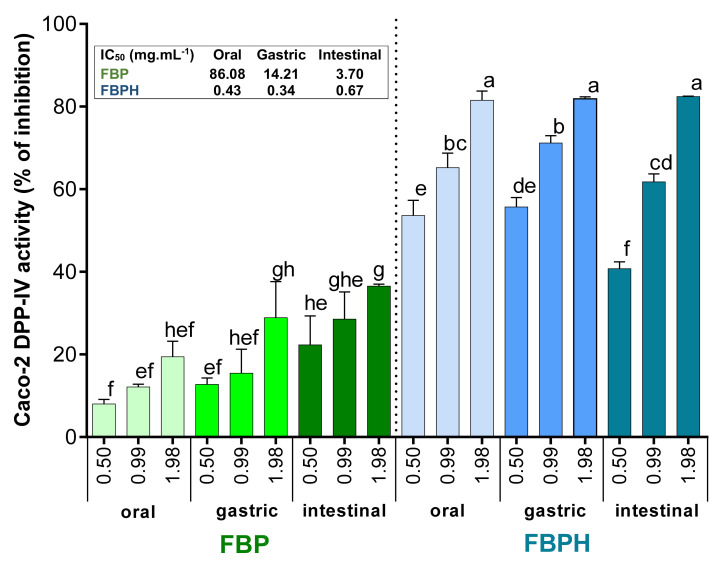
FBPH and FBP effects on the Caco-2 DPP-IV activity inhibition during SGID. The Caco-2 DPP-IV activity inhibition (%) obtained with FBP and FBPH digests assayed at increasing concentrations (0.5; 0.99 and 1.98 g L^−1^, *w*/*v*). Means without a common letter within the same graph are significantly different (*p* < 0.05) using one-way ANOVA followed by Tukey post-hoc test for pairwise comparisons. Inset: IC_50_ were determined by linear regression correlating the DPP-IV activity inhibition percentage and the Ln of the concentration.

The Caco-2 cells exposure to increasing concentrations (from 0.5 to 1.98 mg mL^−1^, *w*/*v*) of oral, gastric and intestinal digests of FBP and FBPH induced a dose-dependent inhibition of the Caco-2 DPP-IV activity. The DPP-IV inhibitory activity potential of FBP increased through the different phases of the SGID. Indeed, the DPP-IV inhibitory activity observed with FBP digests assayed at 1.98 mg mL^−1^ (*w*/*v*) was 1.9-fold higher for intestinal digest than for oral sample. Moreover, the calculated IC_50_ for the intestinal digest (IC_50_ = 3.70 mg mL^−1^) is about 23-fold lower than for the oral sample (IC_50_ = 86.08 mg mL^−1^) (Figure 3). For FBPH, the percentage of DPP-IV inhibitory activity of the samples collected in the three SGID compartments reached approximately 80% at 1.98 mg mL^−1^ (*w*/*v*). The calculated IC_50_ was very closed for the oral, gastric and intestinal digests (insert of Figure 3). This highlights the small effect of the SGID on the DPP-IV inhibitory activity of FBPH. Results also showed that the intestinal digest of FBPH was much more potent than the FBP one. Thus, at a concentration of 1.98 mg mL^−1^ (*w*/*v*), the DPP-IV activity inhibition was about 2.3-fold higher for FBPH than for FBP with a 5.5-fold lower calculated IC_50_ (Figure 3).

### 2.4. CCK and GLP-1 Secretion-Stimulating Peptides Identification

#### 2.4.1. SEC and RP-HPLC Fractionation of FBPH Intestinal Digest

**Figure 4:**
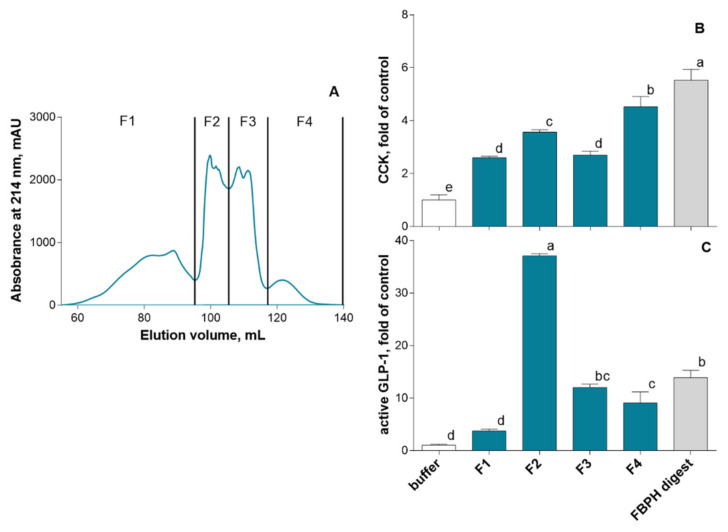
FBPH intestinal digest SEC fractionation effects on gut hormones release in STC-1 cells. The SEC fractionation of the FBPH intestinal digest was performed using a HiLoad 16/600 Superdex prepgrade column with an isocratic gradient of 30% acetonitrile 0.1% TFA (**A**). The amounts of intestinal hormones in the supernatants, after 2 h of contact with the fractions and or the FBPH digest (0.5% *w*/*v*), were determined by radioimmunoassay for CCK (**B**) and active GLP-1 (**C**). Values are the means of three repeated measurements and are expressed in fold of control (buffer) ± SD. Means without a common letter within the same graph are significantly different (*p* < 0.05) using one-way ANOVA followed by a Tukey post-hoc test for pairwise comparisons.

To identify active peptides able to stimulate the secretion of intestinal hormones, we first performed the SEC fractionation of the FBPH intestinal digest (Figure 4A). Four fractions were recovered and put in contact with STC-1 cells at 5 mg mL^−1^ (*w*/*v*) for 2 h. Results obtained showed that all of them were able to stimulate CCK secretion with the F2 and F4 fractions that displayed the higher potential with 3.5- and 4.5-fold of the control CCK secretion level, respectively (Figure 4B). Regarding GLP-1, the F2, F3 and F4 fractions were able to stimulate its secretion in STC-1 cells. The F2 fraction exerted a broadly higher potential than other fractions with 37 FOC and 2.6-fold of the FBPH digest (Figure 4C).

**Figure 5:**
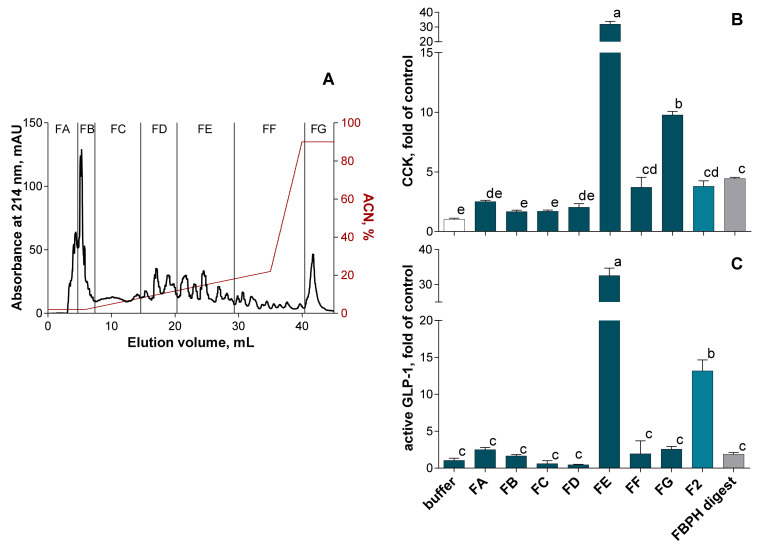
SEC-F2 RP-HPLC fractionation effect on gut hormones release in STC-1 cells. The RP-HPLC fractionation of the FBPH intestinal digest’s SEC-F2 fraction was performed using a C18 Gemini column (150 × 10 mm, particles of 5 μm, 110 Å, Phenomenex) with an ACN gradient represented in red (**A**). The amounts of intestinal hormones released in the supernatants, after 2 h of contact, with the subfractions, the SEC-F2 fraction and the FBPH digest (0.5% *w*/*v*), were determined by radioimmunoassay for CCK (**B**) and active GLP-1 (**C**). Values are means of three repeated measurements and are expressed in fold of control (buffer) ± SD. Means without a common letter within the same graph are significantly different (*p* < 0.05) using one-way ANOVA following by Tukey post-hoc test for pairwise comparisons.

The F2 fraction was thus selected to be fractionated by RP-HPLC on a C18-column and 7 subfractions were designed (Figure 5A). The subfraction FE presented the higher potential to stimulate both CCK (Figure 5B) and GLP-1 (Figure 5C) secretion in STC-1 cells compared with other subfractions. Indeed, the CCK secretion stimulation by the FE subfraction was 31.9-, 8.4- and 7.2-fold higher than those obtained with the buffer, the F2 fraction and the FBPH intestinal digest, respectively. In the same way, the GLP-1 secretion stimulation by the FE subfraction was 32.0-, 2.5- and 17-fold higher than those obtained with the buffer, the F2 fraction and the FBPH intestinal digest, respectively.

#### 2.4.2. RP-HPLC-MS/MS Peptides Identification in the FE Subfraction

**Figure 6:**
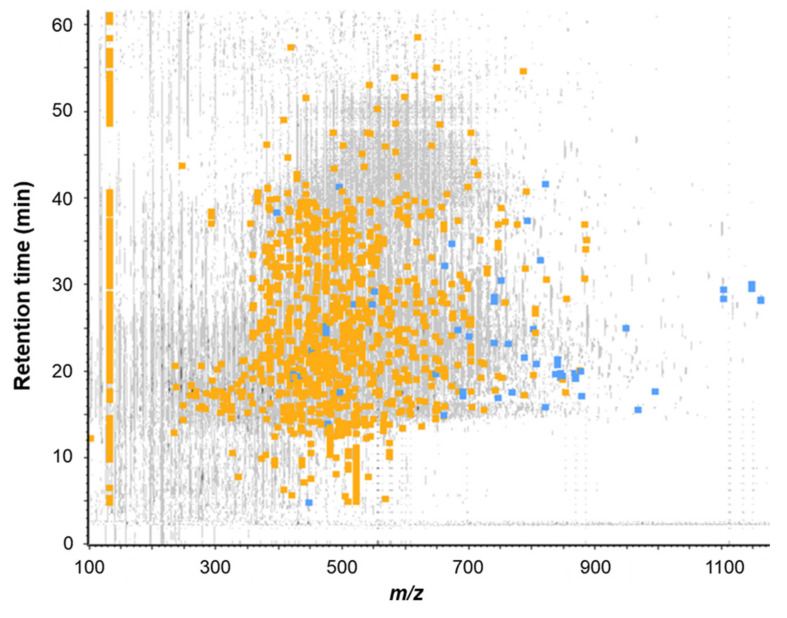
Mass signal 3D-map of the FE subfraction issued from the RP-HPLC separation of the FBPH intestinal digest’s SEC-F2 fraction. Peptide map showing the repartition of all peptides detected by RP-UPLC-MS/MS analysis according to their retention time during chromatography, their mass to charge ratio (*m*/*z*) and their intensity. Grey signals represent all ions detected, blue squares represent identified peptides by database confrontation (false discovery rate (FDR) < 1%) and orange squares represent peptides sequenced by de novo mode (ALC score > 80%).

The FE subfraction was then subjected to RP-HPLC-MS/MS analysis to identify peptides present in this fraction. The Figure 6 showed the mass signal 3D-map obtained.

A total of 1739 peptide sequences were identified (database + de novo with ALC > 80%, data not shown). Among all the identified peptides, 20 of them were selected on the basis of (i) their presence in the most intense peaks of the UV chromatogram (λ = 214 nm), (ii) their ion intensity and (iii) their ion fragmentation quality (Table 1). Those peptides were then chemically synthesized and their ability to stimulate CCK and GLP-1 secretion in STC-1 cells was further assayed.

#### 2.4.3. CCK and GLP-1 Secretion Stimulation Induced by Synthetic Peptides from FBPH Intestinal Digest

**Figure 7:**
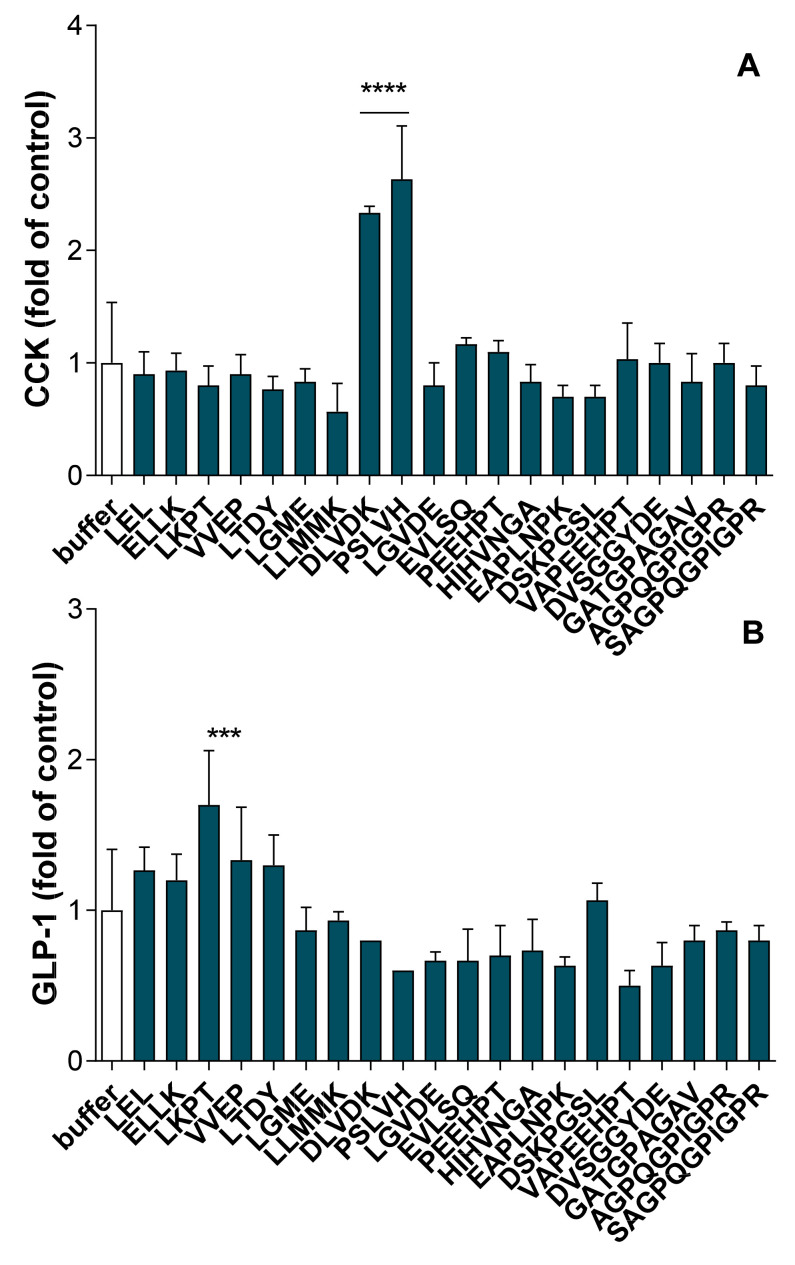
Synthetic peptide effects on intestinal hormones release in STC-1 cells. The amounts of intestinal hormones released in the supernatants, after 2 h of contact with the peptides (1 mM), were determined by radioimmunoassay for CCK (**A**) and active GLP-1 (**B**). Values are means of three repeated measurements and are expressed in fold of control (buffer) ± SD. Means were compared to control mean using one-way ANOVA following by a Dunnett post-hoc test, **** *p* < 0.0001; *** *p* < 0.001.

The synthetized peptides were put in contact with STC-1 cells for 2 h at a final concentration of 1 mM and the amounts of secreted CCK and GLP-1 were further determined by radioimmunoassay. As shown in Figure 7A, among the 20 peptides assayed almost two of them, DLVDK and PSLVH, were able to significantly stimulate CCK secretion (*p* < 0.0001). Regarding GLP-1, only the LKPT peptide was able to significantly stimulate active-GLP-1 release (*p* < 0.001) (Figure 7B).

### 2.5. Identification of Peptides in the Basolateral Side of the Intestinal Barrier Able to Inhibit In Vitro and In Situ the DPP-IV Activity

After 2 h of contact of the FBPH intestinal digest with the Caco-2 cell monolayer in vitro IB model (apical side), 17 peptide sequences were identified by RP-UPLC-MS/MS in the basolateral side. Among these peptides, based on their presence in the most intense peaks of the UV chromatogram monitored at a wavelength of 214 nm, 13 were chemically synthesized, and their DPP-IV inhibitory activity assayed in vitro and in situ (Table 2). Results showed that five peptides (GPFPLLV, VAPEEHPT, VADTMEVV, DPLV and FAMD) were able to inhibit the DPP-IV activity in vitro, with IC_50_ values ranging from 263 to 775 µM. Seven peptides (GPFPLLV, MDLP, DLDL, FAMD, VADTMEVV, CSSGGY and VAPEEHPT) were able to inhibit the in situ DPP-IV activity with IC_50_ values ranging from 456 to 2268 µM. Four peptides (GPFPLLV, VAPEEHPT, VADTMEVV and FAMD) were able to both in vitro and in situ inhibit the DPP-IV activity.

## 3. Discussion

The first goal of this work was to study and to compare the effects of the dog gastrointestinal digestion of a tilapia byproduct protein hydrolysate and its raw material on in vitro cellular markers related to food intake and glucose homeostasis. Consequently, we first developed a static in vitro simulated dog gastrointestinal digestion based on the consensual INFOGEST protocol and on a previously one developed to study protein digestion and, according to previous works performed to investigate drug behavior [25,26] and nutrient digestibility [27] in dogs. As expected, the digestive enzymes (pepsin followed by pancreatin) exerted a more significant impact on the FBP peptide profiles than on those of FBPH, because of industrial enzymes previously digested the raw material. Although the peptide profiles and the apparent MW distribution of FBPH and FBP were quite similar at the end of the SGID, results obtained on intestinal bioactivities highlighted the added benefit of the raw material pre-hydrolysis. Indeed, the FPBH intestinal digest led to a better stimulation of active GLP-1 secretion (44.9 against 29.4 FOC) and a better inhibition of the in situ Caco-2 DPP-IV activity (5.5-fold lower IC_50_ value). Previous results obtained after the SGID of cuttlefish viscera byproduct hydrolysates and their raw material had already showed the added value of the pre-hydrolysis on the recovered intestinal digest DPP-IV inhibitory activity and GLP-1 secretion stimulation [17]. However, this was not the case for CCK secretion stimulation for which the FBPH intestinal digest was slightly less potent than the FBP one (6.2 against 7.9 FOC). The results also highlighted the crucial role of pancreatic enzymes in the apparition of protein-derived peptide bioactivities related to food intake and glucose metabolism regulation. This corroborates results obtained in previous works dealing with the SGID digestion of bovine hemoglobin and sepia byproducts on CCK and GLP-1 secretions in STC-1 cells and DPP-IV activity inhibition [17,28]. In the same way, previous works showed that intestinal digests of casein or bovine hemoglobin induced a higher stimulation of GLP-1 secretion than hydrolysate before SGID [29]. In contrast, hydrolysates may also lose their bioactivities during the gastrointestinal digestion as evidenced for a salmon skin gelatin hydrolysate which lost its GLP-1 stimulatory activity and had a significantly lower DPP-IV inhibitory activity after the SGID [30].

The FBPH intestinal digest exerted a DPP-IV inhibitory potential characterized by an IC_50_ value equal to 1.52 mg·mL^−1^ when obtained by in vitro biochemical test. This is in line with numerous studies showing IC_50_ values for marine byproduct hydrolysates ranging from 1 to 5 mg·mL^−1^ [17,19,22,31,32] and even less than 1 mg·mL^−1^ as for *Gadus chalcogrammus* gelatin [33] and *Salmo salar* hydrolysates [30]. Here, we used an in situ DPP-IV activity test using live Caco-2 cells, which mimics the intestinal environment and, in particular, the enzymatic action of peptidases produced by the epithelial cells of the intestinal brush border [34]. The IC_50_ value obtained for the FBPH intestinal digest was 0.67 mg·mL^−1^. This value is obviously not comparable with those obtained with the in vitro classical test. Nevertheless, this DPP-IV inhibitory activity appears very promising when compared with the IC_50_ (1.57 mg·mL^−1^) obtained for an intestinal cuttlefish byproduct hydrolysate digest in a previous work with the same Caco-2 in situ test [34].

The in vitro results obtained here can be extrapolated to those previously obtained in vivo with other fish protein hydrolysates on food intake and glycemic managements in healthy mice [20] and rats [16], in high-fat-diet-induced obese mice [35] or diabetic and obese rats and as in several clinical studies [18,21,33,36,37,38].

To identify, from the FBPH intestinal digest, active peptides able to stimulate intestinal hormones secretion by EECs, a methodology built on two different successive separation techniques were adopted Caron, J. et al., 2016 [23,25]. Using a first SEC-purification step, the fraction F2 composed by a majority of peptides characterized by apparent MW ranging from 400 to 1000 Da was selected on the basis of its intestinal hormones release activity and submitted to RP-HPLC. Finally, the FE subfraction obtained after RP-HPLC separation exerted the best stimulating release effect for both intestinal hormones, unambiguously.

Among the 1739 peptide sequences identified by the mass bioinformatics data processing, 20 of them were selected (based on their presence in the most intense peaks of the UV chromatogram (λ = 214 nm) and their ion intensity and fragmentation quality), chemically synthesized and assayed for their capacity to stimulate CCK and GLP-1 release. The results allowed to identify two new peptides, PSLVH and DLVDK, able to enhance CCK release by EECs, and one tetrapeptide, FAMD, able to stimulate GLP-1 release. Today, few peptide sequences are reported in the literature to stimulate CCK and GLP-1 secretion by EECs, and the relationship existing between the CCK and GLP-1 food-derived releasing peptide bioactivity and their structure and amino acid sequence is not well established [9,38]. Nevertheless, different signaling pathways, involving G protein-coupled receptors (GPCRs) like GPR93, GPRC6A and the calcium-sensing receptor (CaSR) but also the cotransporter PepT-1, have been evidenced in the food protein-derived peptide intestinal sensing leading to CCK and GLP-1 secretion [39,40,41]. CCK releasing food-derived peptides were identified from soybean β-conglycinin, bovine hemoglobin, lactoglobulin, bovine whey, casein, and egg white protein [42,43,44,45,46,47,48]. To our knowledge, it is the first time from fish source. The motif and the structure of the peptides appear crucial in the intestinal sensing leading to CCK secretion. CaSR was described to sense W and F aromatic amino acids [49,50] and the presence of aromatic residues in the peptide sequence seems to favor the bioactivity. In previous works, we evidenced two CCK-releasing fractions of a bovine hemoglobin SGID intestinal digest. They were able to highly stimulate CCK secretion and composed of more than 50% of peptides containing at least one aromatic amino acid residue in their sequence. The four hemorphins (LLVVYPWT, LVVYPWT, VVYPWT and VVYPWTQRF), released during bovine hemoglobin digestion, were synthesized and proved as CCK and GLP-1 secretion stimulating peptides [42,44]. However, in the present work, the two identified CCK-releasing stimulating peptides, PSLVH and DLVDK, do not possess aromatic residues, whereas DVSGGYDE did not stimulate CCK secretion. These two active peptides are both composed of five amino acid residues and some of them contain an aliphatic chain. These findings are in accordance with a precedent work which hypothesized that five amino acid residues were the minimal size and that the presence of aliphatic chain could be crucial in the CCK secretion in STC-1 cells [47]. In accordance, in a recent work, two peptides able to stimulate CCK secretion in STC-1 cells, VLLPDEVSGL and VLLPD, were identified from an egg white SGID intestinal digest. They both did not contain aromatic amino acid residues but are characterized by a high rate of aliphatic ones [48].

Like for CCK, only few food-derived peptides, able to stimulate the secretion of GLP-1, were identified. We previously identified four sequences (KAAVT, TKAVEH, ANVST and YGAE) from a bovine hemoglobin intestinal digest and proposed that the presence of basic amino acid residue (L-lysine) in the N-terminal side of the peptide, as well as the presence of a T residue in the C- or N-terminal, are common points that could be implied in the peptide sensing that led to GLP-1 secretion [42]. LKPT evidenced in the present work also possesses a lysine amino acid residue in N-terminal position as it was also found in the minimal sequence from α-actinin-2 (KPYIL) able to stimulate the GLP-1 secretion in murine GLUTag cells. However, the K residue position in the sequence does not seem crucial for the bioactivity as ASDKPYIL is also active [51]. The RVASMASEKM peptide, recently identified from egg white protein digest as GLP-1 secretagogue, also possesses a K residue but in C-terminal position [48]. Nevertheless, results obtained here showed that ELLK and EAPLNPK did not lead to GLP-1 secretion, and other works identified peptides able to stimulate GLP-1 secretion without having K or T residues in their sequences, such as GGGG, AAAA, GWGG [52], GPVRGPFPIIV [53], LGG and GF [54] and, PFL [48].

Taken together, these findings confirm the presence of multiple pathways involved in the intestinal peptide sensing leading to CCK and GLP-1 secretion by EECs. It will be necessary to identify which one is used by each peptide to elucidate the relationships between the physicochemical properties, the structure, and the sequence of the peptide and its secretagogue activity.

Among the 13 synthesized peptides identified in the basolateral side of the intestinal barrier model, 5 exerted an in vitro DPP-IV inhibitory activity, with IC_50_ values ranging from 263 to 775 µM (Table 2). These peptides are promising compared with food-derived DPP-IV inhibitory peptides identified between 2016 and 2018 as recently reviewed by Liu et al. [55]. Indeed, when we perform from the Liu et al. list, the analysis of 74 peptides, identified and characterized by IC_50_ values ranging from 43 to 2000 µM, the IC_50_ mean and median values were 596 and 226 µM, respectively. The large majority of the studies which identified DPP-IV inhibitory dietary protein-derived peptides has used in vitro controlled method to calculate IC_50_ values and did not assay the ability of the peptides to cross the intestinal barrier. In the recent work of Harnedy et al., the authors evaluated the DPP-IV inhibitory activity potential of peptides identified from two RP-HPLC fractions of a boarfish hydrolysate submitted to SGID. Several peptides were then synthesized and assayed for their in vitro inhibitory DPP-IV activity. The most promising peptides (IC_50_ < 200 µM) were further assayed for their ability to inhibit in situ the human DPP-IV activity using culture Caco-2 cells in order to better mimic intestinal physiological conditions. They indeed identified 18 peptides with in situ IC_50_ values ranging from 44 to 307 µM [32]. Despite these very interesting findings, the ability of the identified peptides to cross the IB still needs to be studied. Indeed, several studies showed that many DPP-IV inhibitory peptides identified in the intestinal tract cannot cross the IB without being cleaved and losing their bioactivities. Other studies showed that certain DPP-IV inhibitory peptides were able to cross the IB in vitro. Domenger et al. showed that among five DPP-IV inhibitory peptides identified in a bovine hemoglobin intestinal digest, only three of them were recovered intact after the passage through a Caco-2 cells monolayer [44,56]. Lacroix et al., also evidenced the susceptibility of certain milk protein-derived DPP-IV inhibitory peptides to be cleaved by brush barrier peptidases [57]. Indeed, the differentiated Caco-2 cells express mainly two peptidases, DPP-IV and transmembrane protease serine 4 (TMPRSS4) which have been evidenced to hydrolyze peptides during the passage through the simulated IB [58].

In the present study, we adopted the strategy to first incubate the whole digested hydrolysate at the apical side of the IB model for 2 h in order to further identify the peptides in the basolateral compartment. Adopting this strategy also permitted to mimic the interaction of the whole digest with the IB which may modify its permeability. There is some evidence that food-derived peptides could alter intestinal barrier permeability via their actions on tight junction proteins [59,60]. Indeed, we evidenced in a previous work, four hemorphins harboring DPP-IV inhibitory activity able to significantly decrease mRNA expression of the claudin 4, a protein present in tight junctions and involved in paracellular permeability [56]. Finally, the eight new DPP-IV inhibitory peptides which were evidenced in this study might be able in vivo to reach the plasmatic compartment in a sufficient concentration and to inhibit the DPP-IV circulating form, enhancing the half-life of the GLP-1 and therefore its incretin and satiating actions. Moreover, it is crucial to keep in mind that a substantial number of potentially bioactive peptides, in particular small ones, are unidentifiable due to the current peptidomics advance [61]. Further in vivo studies are needed to evidence the glucose and/or food intake regulatory effect of this FBPH, nevertheless, the present tilapia byproduct hydrolysate appears to be very promising as functional ingredient preventing or managing overweight and glucose tolerance.

## 4. Materials and Methods

### 4.1. Materials and Chemicals

Porcine pepsin (EC number 3.4.23.1, from porcine gastric mucosa, >250 U mg^−1^), pancreatin from porcine pancreas (4× USP specifications, European commission number: 232-468-9), diprotin A, Gly-Pro-7-amido-4-methylcoumarin hydrobromide (H-Gly-Pro-AMC, HBr) were purchased from Sigma-Aldrich (Villefranche-sur-Saône, France). Dulbecco’s modified Eagle’s cell culture Medium (DMEM), trypsin, L-glutamine, fetal bovine serum, antibiotics (penicillin and streptomycin) were purchased from Dutscher (Issy-les-Moulineaux, France). Fish byproduct protein hydrolysate (FBPH) was obtained by the hydrolysis of tilapia (*Oreochromis niloticus*) byproducts (FBP, bones and viscera), using commercially available food grade enzymes. FBPH and FBP were provided by Diana Pet Food (Elven, France). Synthetic peptides were purchased from GeneCust (Boynes, France).

### 4.2. In Vitro Simulated Canine Gastrointestinal Digestion of FBP and FBPH

The simulated gastrointestinal digestion (SGID) was adapted from the static in vitro consensual protocol coming from the INFOGEST cost action (http://www.cost-infogest.eu), as well as from Caron et al. in order to mimic the dog gastrointestinal digestion [24,28]. Briefly, the three first steps of the digestive tract (oral, gastric and intestinal) were simulated using a static mono compartmental process and under constant magnetic stirring in a reactor at 39 °C. Two grams of FBPH or FBP were solubilized in 16 mL of salivary fluid at pH 7.0 without salivary enzyme. A 4 mL aliquot (oral aliquot) was withdrawing after 2 min. Twenty-four mL of gastric fluids were then added before the addition of porcine pepsin in a 1:40 (*w*/*v*) E/S ratio (enzymatic activity > 2000 U mg^−1^ of dry weight). Gastric digestion was performed over 2 h, pH being monitored and maintained at pH 2.0 with NaOH (5 M) and HCl (5 M) solutions. Hydrolysate aliquots (gastric aliquots) were withdrawing after 2 h and directly heated at 95 °C during 10 min. Thirty-six mL of intestinal fluid and 4 mL of 1 M NaHCO_3_ solution were added to reach the pH to 6.8. Pancreatin was added in a 1:50 (*w*/*v*) ratio E/S (enzymatic activity 100 U mg^−1^ of dry weight) and intestinal digestion was carried out over 4 h. Aliquots (intestinal aliquots) were withdrawn and heated as above. All aliquots were then centrifuged at 13,000× *g* for 10 min and supernatants were collected to be stored at −20 °C for further analysis.

### 4.3. Size Exclusion Chromatography by Fast Protein Liquid Chromatography (SEC-FPLC)

The peptide apparent molecular weight (MW) distributions of oral, gastric and intestinal aliquots were obtained by SEC using a Superdex Peptide 10/300 GL column (GE Healthcare, Uppsala, Sweden) on an AKTA Purifier system (GE Healthcare). SEC was carried out in isocratic conditions with an elution solution of 30% acetonitrile, 69.9% ultrapure water and 0.1% TFA solvent at a flow rate of 0.5 mL.min^−1^. Oral, gastric and intestinal aliquots were first diluted in ultrapure water (18.5 g L^−1^, *w*/*v*) and subjected to a magnetic stirring for 15 min. The diluted samples were then centrifuged at 15,000× *g* for 15 min and the supernatants were filtered through a 0.22 µm membrane filter before injection. The absorbance was monitored at 214 nm for 70 min. The column was calibrated with the following standard peptides: cytochrome C (12,327 Da), aprotinin (6511 Da), insulin beta-chain (3496 Da), neurotensin (1673 Da), substance P (1348 Da), substance P fragment 1–7 (900 Da) and leupeptin (463 Da).

### 4.4. Cell Culture Conditions

The Caco-2 cell line was purchased from Sigma-Aldrich (Villefranche-sur-Saône, France) and the STC-1 cell line was a grateful gift received from Corinne Grangette (Univ. Lille, CNRS, Inserm, CHU Lille, Institut Pasteur de Lille, U1019-UMR 8204-CIIL, France). Cells were grown in flask of 75 cm^2^ at 37 °C, 5% CO_2_ atmosphere in DMEM supplemented with 4.5 g L^−1^ of glucose, 10% of fetal bovine serum, 100 U mL^−1^ of penicillin, 100 µg mL^−1^ of streptomycin and 2 mM of L-glutamine. Caco-2 and STC-1 cells were weekly and twice a week subcultured, respectively. All cells used in this study were between the 40 and the 50 passages for Caco-2 cells and between the 10 and 30 passages for STC-1 cells.

### 4.5. CCK and GLP-1 Secretion Study

When 80–90% confluence was reached, STC-1 cells were trypsinized and seeded at a density of 40,000 cells/well in 24-wells culture plates (ThermoFisher Scientific, Saint Aubin, France) allowing to reach 60–80% confluence. Cell culture medium was removed from each well and cells were washed with phosphate saline buffer (PBS, 10 mM, pH 7.4). 250 µL of digests (2 to 10 mg mL^−1^) or synthetic peptides (1 mM) diluted in Hepes buffer (4.5 mM KCl, 1.2 mM CaCl_2_, 140 mM NaCl and 20 mM Hepes, pH 7.4) were then added. Hepes buffer was used as a negative control. After 2 h of incubation at 37 °C, 5% CO_2_ atmosphere, supernatants were collected on ice, centrifuged (1500× *g* for 5 min) and stored at −20 °C for further CCK and GLP-1 concentration measurements using GASK-PR (Cisbio, Codolet, France) and GLP-1 active (Merck, Molsheim, France) RIA kits, respectively.

### 4.6. DPP-IV Activity Assay

In situ method using confluent Caco-2 cells described by Caron et al. was slightly modified and used to study DPP-IV activity [34]. A 1 mM (Gly-Pro-AMC) substrate solution, the digests and the synthetic peptides dilutions were prepared in phosphate saline buffer pH 7.4 (PBS). Briefly, after 7 days of growth, Caco-2 cells were trypsinized and seeded at a density of 8000 cells/well in 96-well optical black plates (Nunc, ThermoFisher Scientific, Rochester, NY, USA). After 7 days, culture media were removed from wells and the cells were washed with 100 µL of PBS buffer (pH 7.4). Then, 100 µL of PBS was added to the wells followed by 25 µL of digests diluted in PBS at increasing concentrations (3.47, 6.95 and 13.89 mg mL^−1^) or 25 µL of synthetic peptides diluted in PBS at increasing concentrations (between 0.2; 0.6; 1 and 1.5 mM) or PBS buffer (control wells). After 5 min of incubation at 37 °C, 50 µL of (Gly-Pro-AMC) substrate solution were added to each well. Fluorescence was recorded every 2 min for 1 h at 37 °C using a Xenius XC spectrofluorometer (Safas Monaco, Monaco). Excitation wavelength was set to 260 nm while the emission wavelength was of 480 nm. The percentage of the DPP-IV activity inhibition was defined as the percentage of DPP-IV activity inhibited by a given concentration of digest or diprotin A (commercial DPP-IV peptide inhibitor) as positive control compared with control buffer response. The concentration of digests or synthetic peptide solutions required to obtain 50% inhibition of the DPP-IV activity (IC_50_) was determined by plotting the percentage of DPP-IV activity inhibition as a function of digest or peptide final concentration natural logarithm. IC_50_ was expressed in mg mL^−1^ or in mM.

### 4.7. Fractionation of the FBPH Intestinal Digest

#### 4.7.1. SEC-FPLC Fractionation

The intestinal peptide population of FBPH duodenal SGID was separated on an Akta Purifer device using a preparative HiLoad 16/600 Superdex 30 prepgrade column (GE Healthcare). A volume of 2 mL at 18.5 mg mL^−1^ dry matter of the intestinal digest was injected in the column and eluted in isocratic conditions with an eluent composed of 30% acetonitrile, 69.9% of ultrapure water and 0.1% of TFA at 1 mL.min^−1^ for 2 h. The collected fractions were then dried by centrifuge evaporation (MiVac Quattro Concentrator, Biopharma Process Systems, Winchester, UK) and the obtained pellets were re-solubilized in 1 mL of ultrapure water and stocked at −20 °C.

#### 4.7.2. HPLC Fractionation

The FPLC fractions displaying the strongest bioactivities was fractionated with a semi-preparative C18 Gemini column (150 × 10 mm, particles size 5 μm, 110 Å, Phenomenex, Le Pecq, France) on a 4250 Puriflash system (Interchim, Montluçon, France). The peptide elution was performed at a flow rate of 5 mL.min^−1^ with two solvents: eluent A was composed of 99.9% of ultrapure water and 0.1% TFA and eluent B was composed of 99.9% of acetonitrile and 0.1% TFA. The following hydrophobic gradient was used: an isocratic step at 98% of eluent A for 20 min followed by a linear gradient from 2% to 15% of eluent B in 35 min, then a linear gradient from 15% to 90% of eluent B in 10 min and finally the column was washed with 90% of eluent B for 5 min and equilibrated again with 98% of eluent A for 10 min. The collected subfractions were then dried by centrifuge evaporation (MiVac Quattro Concentrator, Biopharma Process Systems).

### 4.8. Peptide Sequences Identification in HPLC Fractions

#### 4.8.1. RP-HPLC-MS/MS Analysis of HPLC Fractions

Selected dried HPLC subfractions were re-solubilized in 50 μL of ultrapure water containing 0.1% of formic acid (FA), vortexed, submerged in ultrasonic bath three times and finally centrifuged 5 min at 12,000× *g*. The peptides of these fractions (10 μL injection volume) were then chromatographed by reverse phase-ultra high-performance liquid chromatography (RP-UPLC) using an ACQUITY biocompatible chromatography system (Waters, Manchester, UK) equipped with an analytical C18 Uptisphere column (250 × 3 mm, particles size 5 μm, 300 Å, Interchim). The peptides elution was performed at 30 °C with a flow rate of 0.6 mL.min^−1^ using two solvents: eluent A was composed of 99.9% of ultrapure water and 0.1% FA and eluent B of 99.9% of acetonitrile and 0.1% FA. Apolar elution gradient used was: 100% of eluent A for 2 min followed by a linear gradient from 0 to 15% of eluent B in 45 min, then a linear gradient from 15% to 35% of eluent B in 20 min and from 35% to 90% of eluent B in 15 min. The column was finally washed with 90% of eluent B for 10 min and equilibrated with 100% of eluent A for 7 min.

The chromatographed peptides were then ionized into the electrospray ionization source of the qTOF Synapt G2-Si™ (Waters). MS analysis was performed in sensitivity, positive ion and data dependent analysis (DDA) modes. The source temperature was set at 150 °C, the capillary and cone voltages were set at 3000 and 60 V, respectively. MS and MS/MS measurements data were performed in a mass/charge range fixed between 100 to 2000 *m*/*z* with a scan time of 0.2 s. A maximum of 15 precursor ions with an intensity threshold of 10,000 were selected for the fragmentation by collision induced dissociation (CID) with specified voltages ranging from 8 to 9 V and from 40 to 90 V for the lower molecular mass ions and for those with a higher molecular mass, respectively. The leucin enkephalin ([M + H]^+^ of 556.632) was injected in the system every 2 min for 0.5 s to follow and to correct the measure error during all the time of analyze.

#### 4.8.2. Mass Spectrometry Data Processing

Mass spectrometry data processing and the protein database search were performed via Peaks Studio version 8.5 software (Bioinformatics Solutions, Waterloo, ON, Canada) using UniProt database restricted to the complete proteome of the Cichlidae family (updated the 2018/08/28, 44,684 entries). Tolerance threshold of precursor ion masses and fragments were defined at 35 ppm and 0.2 Da, respectively. The in-database identification search was performed with consideration of oxidized methionine but without notifying the choice of enzyme. Peptide sequences identified by the Peaks Studio 8.5 were filtered with a fault discovery rate (FDR) strictly lower than 1% while peptide sequences identified by de novo processing were filtered according to an average local confidence score (ALC score) up to 80%.

### 4.9. Intestinal Barrier Passage and Peptide Identification

#### 4.9.1. Transport Study

To obtain an intestinal barrier (IB) model, Caco-2 cells were cultivated on insert (D = 4.2 cm; pore size = 3 μm, ref: 353092, Dutscher) in 6-wells plate where Caco-2 cells were seeded at a density of 84,000 cells by insert in 2 mL of DMEM. A volume of 2.5 mL of DMEM medium was added in each well of the plate. Cells were incubated at 37 °C for 15 days and the medium on apical and basolateral sides were changed every 2 days.

In the day of experimentation, a transport medium Hepes-Hanks salt solution (HBSS) was extra temporary prepared and filtered on PVDF filter 0.22 μm. Samples were diluted at 4 g L^−1^ with the transport medium. The apical and basolateral sides of each well were washed with 500 μL and 1 mL of transport medium (heated at 37 °C), respectively. Then, 1 mL of transport medium at 37 °C was added in apical side and 2.5 mL in basolateral side. Plate was incubated at 37 °C, 5% of CO_2_ for 30 min, and the supernatant was discarded and replaced with 1 mL of pre-heated samples or pre-heated transport medium (for the control). Kinetic studies were performed by sampling 100 μL from the apical and basolateral sides at 15 min, 250 μL in apical side and 1 mL in basolateral side at 60 min and the rest of the supernatant in apical (650 μL) and in basolateral side (1.4 mL) at 120 min of incubation at 37 °C, 5% CO_2_. The peptides were identified after 120 min incubation time.

#### 4.9.2. Peptide Sequences Identification in Apical and Basolateral Supernatant by Mass Spectrometry

Apical and basolateral supernatants at 120 min were prepared and analyzed by mass spectrometry with the same protocol described above for HPLC fractions with minor changes. The UPLC column used was a C18-AQ (150 × 3 mm, particles size: 2.6 µm, 83 Å, Interchim) and the peptide chromatography was performed at a flow rate of 0.5 mL·min^−1^ and 30 °C. The apolar elution gradient used was as follow: 5 min at 99% of eluent A/1% eluent B, then a linear gradient from 1% to 30% of eluent B in 40 min, followed by a linear gradient from 30% to 70% of eluent B in 8 min, and finally after 2 min at 95% of eluent B, the column was equilibrated with 99% of eluent A/1% eluent B for 3 min. The ionization mode, and the MS and MS/MS measures were performed exactly as described previously.

### 4.10. Statistical Analysis

Data presented are means ± SD. To compare GI hormone secretion levels induced by the digests, a one-way ANOVA using general linear model and pairwise comparisons with Tukey’s or Dunnett’s tests were performed using Graph Prism (GraphPad Software, San Diego, CA, USA). Values were considered as significantly different for a *p*-value < 0.05.

## 5. Conclusions

A dog in vitro static simulated gastrointestinal digestion model that permitted us to evaluate in vitro the potential effects of a tilapia byproduct hydrolysate on the regulation of food intake and glucose metabolism was developed. Promising effects on intestinal hormones secretion and dipeptidyl peptidase IV (DPP-IV) inhibitory activity were thus evidenced and the added-value of the pre-hydrolysis was highlighted. New bioactive peptides able to stimulate CCK (DLVDK and PSLVH) and GLP-1 (LKPT) secretion and to inhibit the DPP-IV activity after a transport study through an intestinal barrier (VAPEEHPT, DLDL, MDLP, VADTMEVV, DPLV, FAMD, CSSGGY and GPFPLLV) were identified. This tilapia byproduct hydrolysate appears to be promising to manage overweight.

## Figures and Tables

**Table 1:** List of the 20 peptides selected for chemical synthesis, following their identification by database confrontation or de novo sequencing. Peptides were listed according to their RP-UPLC-MS/MS characteristics (retention time (RT), mass to charge ratio (*m*/*z*) and their identification score displayed (i) by the average local confidence (ALC) score for the de novo sequencing, (ii) by the −10^logP^ score for database confrontation with the mass error (in ppm) for both identification modes (ID). nd: when the identification of the parent protein was not possible.

**ID Mode**	**Sequence**	**RT**	***m*/*z***	**10^−logP^**	**ppm**	**Identification**
Database	SAGPQGPIGPR	27.11	518.78	46.51	0.6	Collagen type I
	DVSGGYDE	20.69	841.33	41.49	6.2	Collagen type I
	HIHVNGA	16.85	747.39	38.12	−1.5	Collagen type 10
	VAPEEHPT	17.03	879.42	36.41	3.5	Alpha-actin
	AGPQGPIGPR	24.51	475.26	35.37	0.0	Collagen type I
	GATGPAGAV	23.75	700.36	32.09	0.2	Collagen type I Alpha-actin
	EAPLNPK	17.44	768.42	31	−4.6	Alpha-actin
	PEEHPT	16.74	709.32	27.74	8.3	Alpha-actin
**ID Mode**	**Sequence**	**RT**	***m*/*z***	**ALC**	**ppm**	
De novo	LGVDE	21.54	532.26	98	2.6	nd
	VVEP	19.09	443.24	96	−10.4	nd
	LTDY	20.97	511.25	96	15.1	nd
	PSLVH	17.43	552.30	96	−33.1	nd
	ELLK	16.65	502.31	95	−31.5	nd
	LGME	21.28	449.21	95	6.9	nd
	DLVDK	17.39	589.32	95	10.6	nd
	EVLSQ	21.84	575.29	95	−23.8	nd
	LKPT	20.48	235.67	92	−9.1	nd
	DSKPGSL	17.48	703.37	92	6.1	nd
	LLMMK	17.19	318.18	90	−26.9	nd
	LEL	18.83	374.23	80	−1.6	nd

**Table 2:** In vitro and in situ DPP-IV inhibitory activity of the 13selected-chemically synthesized peptides following their identification by database confrontation or de novo sequencing (ID mode). Peptides were listed according to their RP-UPLC-MS/MS characteristics (retention time (RT) and mass to charge ratio (*m*/*z*) and their identification score displayed (i) by the average local confidence (ALC) score for the de novo sequencing, (ii) by the −10^logP^ score for database confrontation with the mass error (in ppm) for both identification modes. Values of the in vitro and in situ DPP-IV inhibitory activity (IC_50_) were determined by linear regression correlating the DPP-IV activity inhibition percentage and the Ln of the peptide concentration. nd: when the identification of the parent protein was not possible and when the IC_50_ value was above 2500 µM or undeterminable.

ID Mode	Sequence	RT	*m*/*z*	ALC	10^−logP^	In Vitro IC_50_ (µM)	In Situ IC_50_ (µM)	Identification
Database	VAPEEHPT	15.47	440.21	28.54	−3.9	409	2268	Alpha-actin
De novo	DLDL	26.88	475.24	98	−6.4	nd	763	nd
De novo	PDLV	20.77	443.24	89	−12.7	nd	nd	nd
De novo	MDLP	26.87	475.24	87	31	nd	605	nd
De novo	VDAGAP	16.52	529.26	84	0.7	nd	nd	nd
De novo	EDYT	27.16	264.10	84	−13.5	nd	nd	nd
De novo	VADTMEVV	16.06	440.21	82	4.3	603	1130	nd
De novo	DPLV	22.33	443.25	81	−8.9	698	nd	nd
De novo	EDTY	27.63	264.10	81	−14.2	nd	nd	nd
De novo	CSSGK	27.77	481.21	80	6.2	nd	nd	nd
De novo	FAMD	13.51	483.19	80	−1.4	775	862	nd
De novo	CSSGGY	29.88	573.20	80	6.4	nd	2160	nd
De novo	GPFPLLV	42.46	742.45	80	2.0	263	456	nf

## Data Availability

The data presented in this study are available on request from the corresponding author. The data are not publicly available due to this study is an industrial work and some results are confidential.

## Sample Availability

Samples of the compounds are not available from the authors.
